# Supplementary material for: Amundsen Sea circulation controls bottom upwelling and Antarctic Pine Island and Thwaites ice shelf melting
Source: Nat Commun. 2024 Apr 11;15:2946. doi: 10.1038/s41467-024-47084-z (PMC11009355; doi:10.1038/s41467-024-47084-z)
Supplement: Supplementary file 1 — Supplementary Information [file 41467_2024_47084_MOESM1_ESM.pdf]

SUPPLEMENTARY INFORMATION

Amundsen Sea Circulation Controls Bottom Upwelling and Antarctic Pine Island and Thwaites Ice Shelf Melting

Taewook Park[1*], Nakayama Yoshihiro[2*], SungHyun Nam[3]

[1]Division of Ocean and Atmosphere Sciences, Korea Polar Research Institute, Incheon 21990, Republic of Korea

[2]Institute of Low Temperature Science, Hokkaido University, Sapporo 060-0819, Japan

[3]School of Earth and Environmental Sciences/Research Institute of Oceanography, Seoul National University, Gwanak-gu, Seoul 08826, Republic of Korea

Correspondence:

*Nakayama Yoshihiro (Yoshihiro.Nakayama@lowtem.hokudai.ac.jp)

*Taewook Park (twpark@kopri.re.kr)

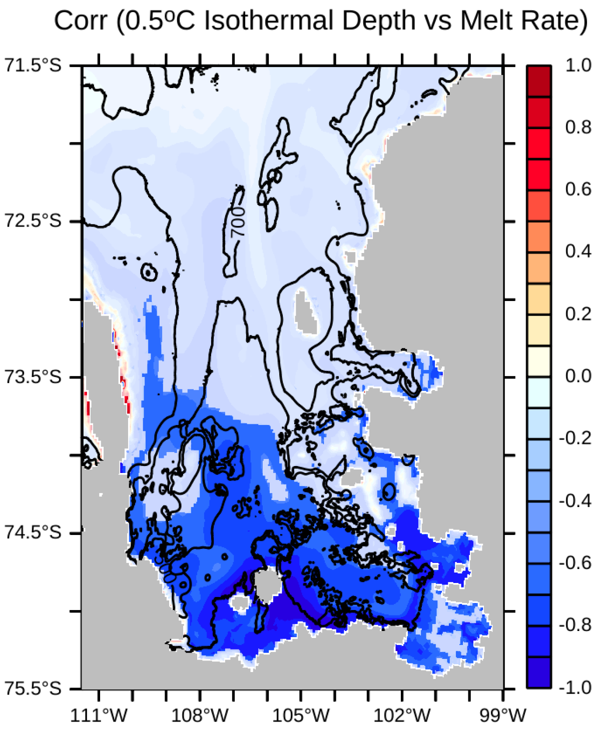

Supplementary Figure 1. Map of correlation coefficients between 0.5°C isotherm depth and the melt rates of both Pine Island and Thwaites Ice Shelves. Statistically insignificant regions in 95%-level are shaded transparently. Black color-coded contours indicate the bathymetry of 500 m and 700 m depth.

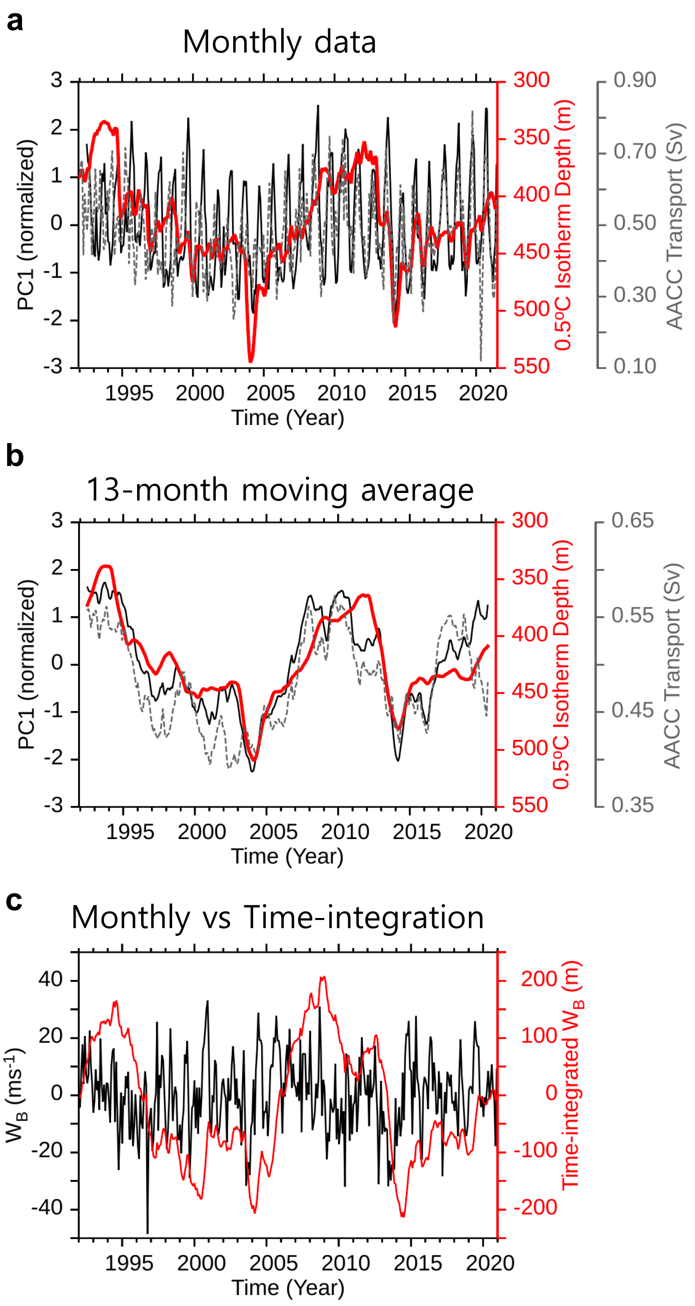

Supplementary Figure 2. **a** The leading mode's principal component (PC1) of ocean current (black line), southward Antarctic Coastal Current (AACC) transport across 74.4°S, 104–101.5°W in Pine Island Bay (grey dotted line), and 0.5°C isotherm depth averaged south of X–X' in Fig. 1a in the main text (red line). All variables are derived from the monthly data. **b** The same as in **a**, but seasonal variations of all variables are removed using a 13-month moving average filter, which is the same as Fig. 2b in the main text. **c** Comparison of the monthly vertical velocity anomaly in the bottom layer (black line) and its time integration (red line). This time-integrated vertical velocity does not differ much from the one with an additional 13-month moving average filter in the main text.

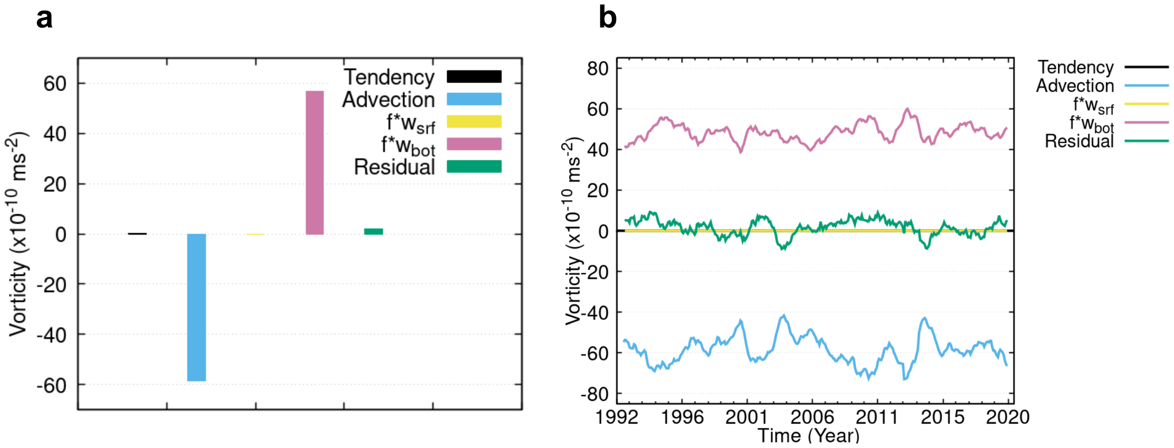

Supplementary Figure 3. **a** Depth-integrated vorticity budget terms (time tendency of vorticity, relative vorticity advection, surface and bottom vortex stretching term, and residual term) averaged south of X–X' in Fig. 1a in the main text. **b** Timeseries of vorticity budget terms. Seasonal variations of all the budget terms are removed using a 13-month moving average filter.

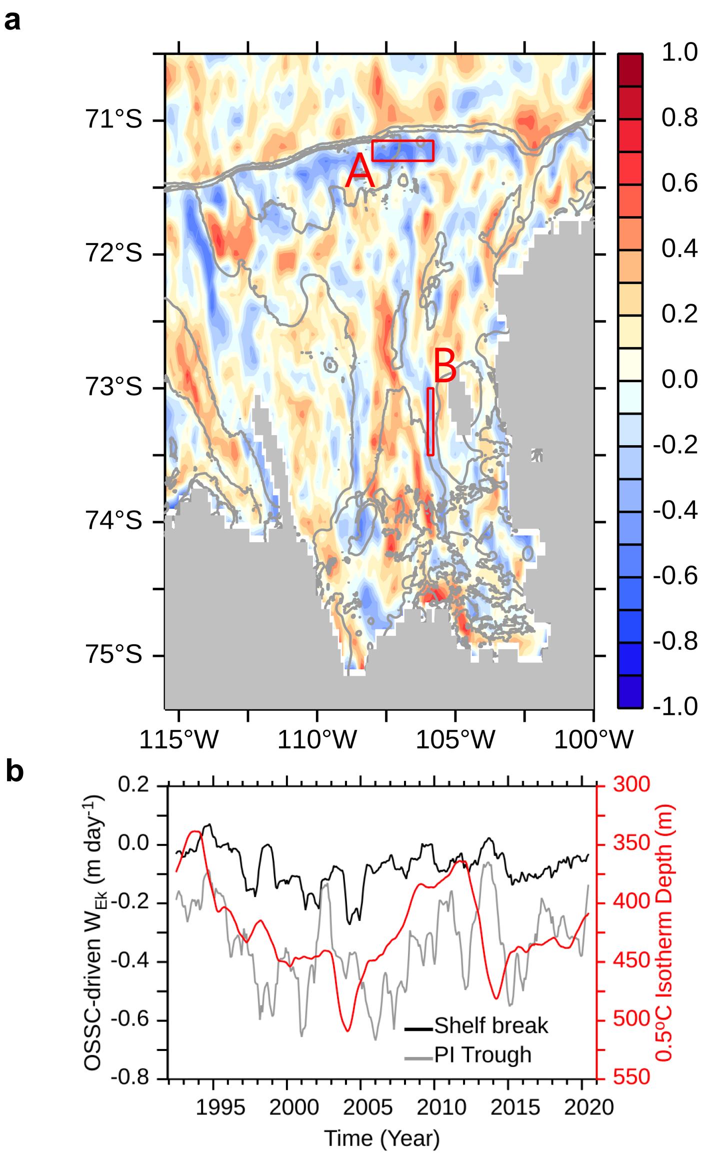

Supplementary Figure 4. **a** Map of correlation coefficients between the spatial distribution of ocean surface stress curl (OSSC)-driven Ekman pumping velocity with positive values indicating upwelling and an index of Pine Island Bay (PIB)-averaged thermocline depth. **b** Black line represents OSSC-driven Ekman pumping velocity averaged in the region A (continental shelf break) in **a**, gray line represents that in Pine Island Trough in **a**, and red line represents PIB-averaged thermocline depth. Box areas were selected in the continental shelf break and Pine Island Trough where there are negative correlations, indicating the Ekman upwelling is associated with TD lifting. A 13-month moving averaged data is used in the calculation.

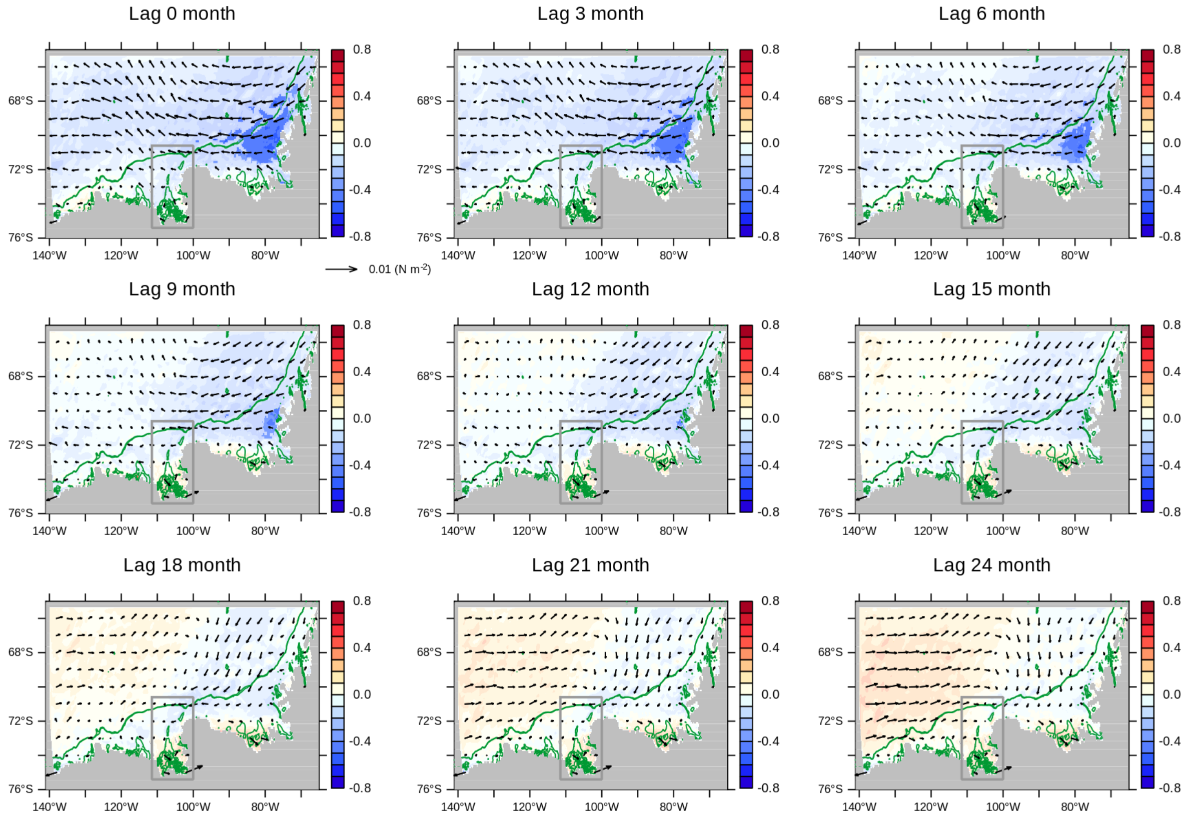

Supplementary Figure 5. Lagged regression coefficients (arrows) of wind anomalies on the leading mode's principal component (PC1) of ocean current (inside the gray-colored box) in the eastern Amundsen Sea, superimposed with their correlation coefficients (color shading) of the zonal wind and the PC1. Statistically insignificant regions in the 90%-level are transparently shaded. The "Lag (month)" denotes a time delay of the ocean circulation in the eastern Amundsen Sea to wind patterns in month. A green color-coded contour indicates the bathymetry of 700 m and 1000 m depth.

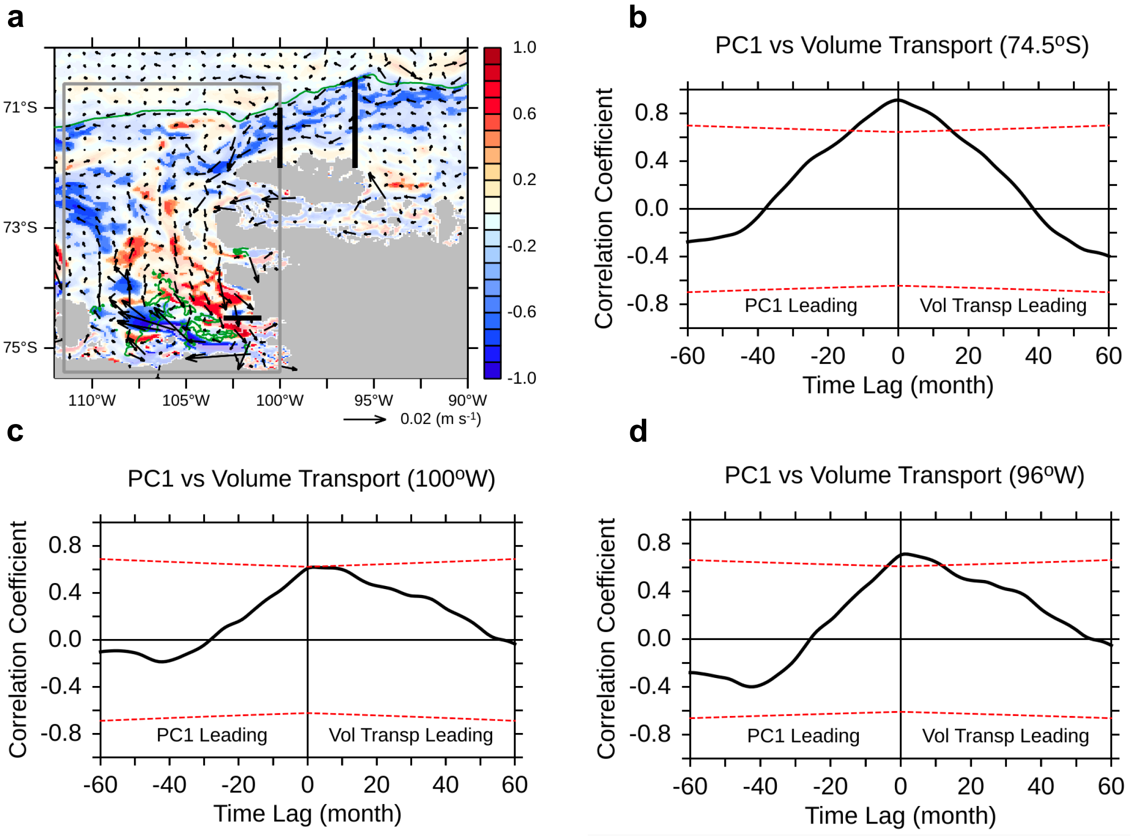

Supplementary Figure 6. **a** Regression coefficients (arrows) of ocean current anomalies averaged above the 0.5°C isotherm depth on the leading mode's principal component (PC1) of ocean current superimposed with their correlation coefficients (color shading) between the PC1 and zonal ocean current. Statistically insignificant regions in 90%-level are shaded transparently. Three sections (thick black line) are for the lead-lag correlation plots in **b**, **c**, **d**. **b**, **c**, **d** Lead-lag correlations (black line) between PC1 and inflow volume transport each section at a 95% confidence level (red dotted line).

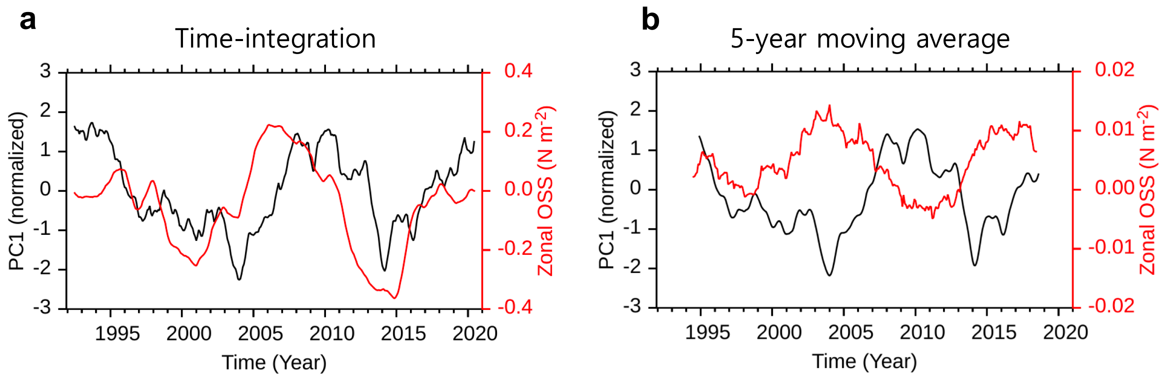

Supplementary Figure 7. The leading mode's principal component (PC1) of ocean current (black line) with **a** time-integrated zonal ocean surface stress (red line) and **b** zonal ocean surface stress after applying 5-year moving average filter (red line). The ocean surface stress is averaged on the continental shelf break within the box A in Fig. 4c in the main text.
